# *CCL3L1* copy number, *CCR5* genotype and susceptibility to tuberculosis

**DOI:** 10.1186/1471-2350-15-5

**Published:** 2014-01-09

**Authors:** Danielle Carpenter, Carmen Taype, Jon Goulding, Mike Levin, Brian Eley, Suzanne Anderson, Marie-Anne Shaw, John AL Armour

**Affiliations:** 1 School of Life Sciences, University of Nottingham, Nottingham NG7 2UH, UK; 2 Institute of Integrative and Comparative Biology, Faculty of Biological Sciences, University of Leeds, Leeds LS2 9JT, UK; 3 Department of Medicine, Imperial College London, South Kensington Campus, London SW7 2AZ, UK; 4 Department of Paediatrics and Child Health, Red Cross War Memorial Children's Hospital, University of Cape Town, Cape Town, South Africa; 5 Medical Research Council Unit, Banjul, Fajara, The Gambia

**Keywords:** *CCL3L1*, *Mycobacterium tuberculosis*, Association, CCR5, MIP-1α

## Abstract

**Background:**

Tuberculosis is a major infectious disease and functional studies have provided evidence that both the chemokine MIP-1α and its receptor CCR5 play a role in susceptibility to TB. Thus by measuring copy number variation of *CCL3L1*, one of the genes that encode MIP-1α, and genotyping a functional promoter polymorphism -2459A > G in CCR5 (rs1799987) we investigate the influence of MIP-1α and CCR5, independently and combined, in susceptibility to clinically active TB in three populations, a Peruvian population (n = 1132), a !Xhosa population (n = 605) and a South African Coloured population (n = 221). The three populations include patients with clinically diagnosed pulmonary TB, as well as other, less prevalent forms of extrapulmonary TB.

**Methods and results:**

Copy number of *CCL3L1* was measured using the paralogue ratio test and exhibited ranges between 0–6 copies per diploid genome (pdg) in Peru, between 0–12 pdg in !Xhosa samples and between 0–10 pdg in South African Coloured samples. The *CCR5* promoter polymorphism was observed to differ significantly in allele frequency between populations (*A; Peru f = 0.67, !Xhosa f = 0.38, Coloured f = 0.48).

**Conclusions:**

The case–control association studies performed however find, surprisingly, no evidence for an influence of variation in genes coding for MIP-1α or CCR5 individually or together in susceptibility to clinically active TB in these populations.

## Background

Tuberculosis (TB), caused by the pathogen *Mycobacterium tuberculosis*, is a leading cause of global mortality and morbidity. The World Health Organisation estimates that one third of the world’s population is infected with TB, with an estimated 1.7 million deaths from TB in 2009.

A good protective immune response against *M. tuberculosis* requires the formation of discrete granulomatous lesions to contain the mycobacteria, and a complex interaction between activated T cells, macrophages and polymorphonuclear leucocytes [1]. The granuloma creates a micro-environment where infected macrophages, dendritic cells and different T-cell populations exist in close proximity and limit *M. tuberculosis* growth and spread [1]. A Th1 response, and in particular interferon γ (IFNγ) production, are essential for the activation of macrophages and the control of infection [2,3]. Tumour necrosis factor (TNF)-α and IFN-γ activate the production of pro-inflammatory cytokines, including TNF-α and in particular macrophage inflammatory protein (MIP)-1α and MIP-1β, which have been shown to play a role in recruitment and activation of macrophages and leucocytes at the sites of infection [4,5].

Previous studies have demonstrated the central role of MIP-1α in the host’s protective immune response to other intracellular pathogens; MIP-1α is essential for the clearance of *Listeria monocytogenes* infection [6], and to activate macrophages critical for the eradication of *Leishmania*[7]. As MIP-1α functions as a chemoattractant for monocytes, macrophages and lymphocytes it may play a crucial role in TB pathogenesis. MIP-1α has been detected in the lungs of mice in response to infection with TB [8], and increased MIP-1α mRNA expression has been observed in humans infected with TB [9,10]. MIP-1α mRNA expression and protein production has been shown to be upregulated by *M. tuberculosis* stimulation, and secretion of MIP-1α was detected during the generation of granulomatous lesions [11]. MIP-1α production was also observed to be higher in HIV patients with pulmonary TB than in controls (patients with HIV but without TB infection) [12].

MIP-1α is a small, low molecular weight, β-chemokine and acts as a pro-inflammatory cytokine [13,14]. MIP-1α is encoded by the genes *CCL3* and the copy number variable paralogous gene *CCL3L1*, located on chromosome 17, which exhibit a high degree (96%) of nucleotide and protein similarity. More specifically, *CCL3* and *CCL3L1* encode MIP-1α isoforms LD78α and LD78β respectively, with the isoform LD78β being 2-fold more efficient at chemoattracting human lymphocytes than the LD78α isoform [15].

MIP-1α is the natural ligand for the C-C chemokine receptor type 5 (CCR5), present on many immune cells including macrophages, T-cells and dendritic cells, and functions in the movement of leucocytes to sites of infection. During *M. tuberculosis* infection expression of CCR5 has been shown to be upregulated [16-18], and this receptor preferentially used [19], suggesting a major role in the trafficking of leukocytes during TB infection. CCR5 is encoded by the gene *CCR5* on chromosome 3, and polymorphisms within *CCR5* have been identified which alter the response of CCR5 to chemokines including alterations to the ligand binding properties of CCR5 [20-22] and a promoter polymorphism in *CCR5* -2459A > G (rs1799987), shown to influence expression levels of *CCR5*[23]. Recently a *CCR5* promoter haplotype, *CCR5-HDD*, known to increase susceptibility to HIV-1, has also been associated with TB infection [24].

The aim of this study is to investigate the influence of genetic variation in the genes coding for MIP-1α and CCR5 in susceptibility to clinically active TB infection in three different populations, Peruvian, !Xhosa and South African Coloured. Copy number variation of *CCL3L1* is investigated, and the functional promoter polymorphism in *CCR5* -2459A > G (rs1799987) is examined.

## Methods

### Study population

This study analyses samples from three populations, a Peruvian population from Lima (n = 1132), and two populations from the Western Cape region of South Africa; an isolated !Xhosa population (n = 605) and a South African Coloured population of mixed ancestry (n = 221) (see Table 1). All gDNA was isolated from whole blood.

**Table 1:** Number of genotyped cases and control for each population

**Population**	**Cases**		**Controls**		**Total**
	**Adult**	**Paediatric**	**Adult**	**Paediatric**	
Peruvian	621	0	511	0	1132
!Xhosa	0	141	341	123	605
Coloured	0	56	152	13	221

The population samples from Peru were collected between 1999 and 2002 from the north of Lima City, with approval of the joint ethics committee of Universidad Peruana Cayetano Heredia and with informed consent and have previously been described [25,26]. In brief the samples consist of adult patients with clinical diagnosis of pulmonary (n = 498), pleural (n = 79), miliary (n = 35) or other extra-pulmonary (n = 9) forms of TB. There were 511 age-matched healthy adult control individuals (age range 32.56 ±9.39 years) from the same geographical area of Lima as the cases and with a similar socioeconomic background and no past history of TB. Patients were not enrolled in the study if presenting with HIV infection, diabetes mellitus or other disease increasing the risk of developing TB.

The two South African population samples, !Xhosa and Coloureds, consisted of paediatric and adult samples and were collected with the approval of the ethics committees of University of Cape Town, South Africa and St Mary’s Hospital, London, UK and with informed consent. The paediatric samples (n = 333) included both !Xhosa and Coloured samples and both TB cases and controls (Table 1) and were all from crowded townships in Cape Town and have previously been described [27]. All the paediatric samples were tested for HIV and found to be negative. Some, but not all of the paediatric cases were clinically diagnosed with pulmonary (!Xhosa n = 55; Coloured n = 24) and extra-pulmonary TB (!Xhosa n = 82; Coloured n = 31). The paediatric controls were all age-matched and from the same neighbourhood as the cases The adult !Xhosa and Coloured samples (n = 493) consisted entirely of control samples and were more stringent controls as there was no history of previous TB disease during childhood and all had a positive Mantoux test to confirm exposure to *M. tuberculosis*.

### Copy number measurement

*CCL3L1* copy number was measured from genomic DNA using the paralogue ratio test (PRT) previously described by Carpenter *et al.*[28,29], modified from the method of Walker *et al.*[30]. Briefly, the PRT method is a PCR based assay using a single pair of primers to simultaneously amplify two specific products in a single reaction, one from a single-copy reference locus and the other from a copy variable test locus of interest [31]. The copy number of the test locus is then estimated from the ratio of test to reference PCR products. The Peruvian samples were measured using a single tube triplex assay [29], whereas the !Xhosa and Coloured samples were measured using a three tube triplex assay with two microsatellite assays [28].

For each sample the products from the PCR reactions were mixed with 10μl HiDi formamide with ROX-500 marker (Applied Biosystems) for analysis. Fragment analysis was carried out by electrophoresis on an ABI3100 36 cm capillary using POP-4 polymer with an injection time of 30s at 2kV.

GeneMapper software (Applied Biosystems) was used to extract the peak areas of the PRT products and the ratio of test to reference amplicons was calculated for each sample independently. Copy number values were calculated by calibrating the ratios; for the Peruvian samples calibration used European ECACC HRC-1 (http://www.hpacultures.org.uk) samples [C0075 with a copy number (CN) = 1; C0150 with CN = 2; C0007 with CN = 3; and C0877 with CN = 4], which were included in every experiment in duplicate, and calibrating the !Xhosa and Coloured samples used 5 Yoruba HapMap samples (http://www.coriell.org) [copy number (CN) =2, NA19159; CN = 3, NA18870; CN = 4, NA19092; CN = 5, NA19171; and CN = 6, NA18503], which were included in every experiment in triplicate. Unrounded and calibrated copy numbers were compared and an average copy number value calculated. For the !Xhosa and Coloured samples the average copy number was then compared with microsatellite data to ascertain integer copy number (see Additional file 1).

### *CCR5* genotyping

The *CCR5* gene contains a number of polymorphisms, some of which have previously shown associations with susceptibility to HIV-1 infection [23,32-34]. In this study we chose to investigate a *CCR5* promoter polymorphism, -2459A > G, that has been shown to influence expression of the CCR5 receptor, such that the -2459*A allele tends to have increased promoter activity [23,35]. Furthermore *in vivo* association studies provide support for this as it has been shown that -2459*AA HIV-1 infection individuals progress more rapidly than those with a -2459*GG genotype [23].

*CCR5* SNP (rs1799987) -2459A > G genotyping was performed by PCR-RFLP using a previously designed method [23]. Briefly the primers (forward 5′–CCC GTG AGC CCA TAG TTA AAA CTC-3′ and reverse 5′-TCA CAG GGC TTT TCA ACA GTA AGG-3′) generate an amplicon of 286bp in length. In the -2459*G allele this is cleaved by *Bsp*1286I to produce 3 fragments of 127bp and 149bp, and 10bp. The second cleavage fails to occur in the presence of the -2459*A allele and so only a band of 276bp is observed, as well as the small 10bp band on a 2% (w/v) agarose gel.

### Statistical analysis

The difference in mean *CCL3L1*/*CCL4L1* copy number between the three population samples was initially assessed using an ANOVA. The distribution of *CCL3L1*/*CCL4L1* copy number in all populations closely follows a normal distribution, and therefore a two-tailed t- test was used to assess differences in the means between cases and controls and between pairs of populations. *CCR5* allele frequencies were calculated manually from genotype frequencies from the different populations using the control samples only. *CCR5* genotype distributions were compared between populations and between patients and controls using a χ^2^ test in SPSS. To investigate the influence of *CCR5* genotype on disease, adjusting for copy number variation, logistic regression was performed in SPSS with *CCL3L1* copy number and *CCR5* genotype as a continuous covariates.

## Results

### Copy number measurements

The distribution of integer copy number for the Peruvian population samples is shown in Additional file 1: Table S1 and suggests a range of 0–6 copies, with a mean of 3. The concordance between PRT measurements is strong, with 96% of samples having all measurements concordant within 0.75 of the assigned integer, and low probability of error as discussed in detail in the Additional file 1.

The distributions of integer copy numbers for the !Xhosa and Coloured samples are shown in Additional file 1: Tables S2 and Table S3 suggesting the !Xhosa samples to have a broad range and mean copy number of 4, whereas the Coloured samples have a mean of 3. In general the concordance between the nine PRT measurements is good and the microsatellite data are in agreement with the consensus integer in 94% of all South African samples. The distribution of unrounded copy number, probability of error and accuracy of the copy number genotyping are all examined in detail in the Additional file 1.

**Figure 1:**
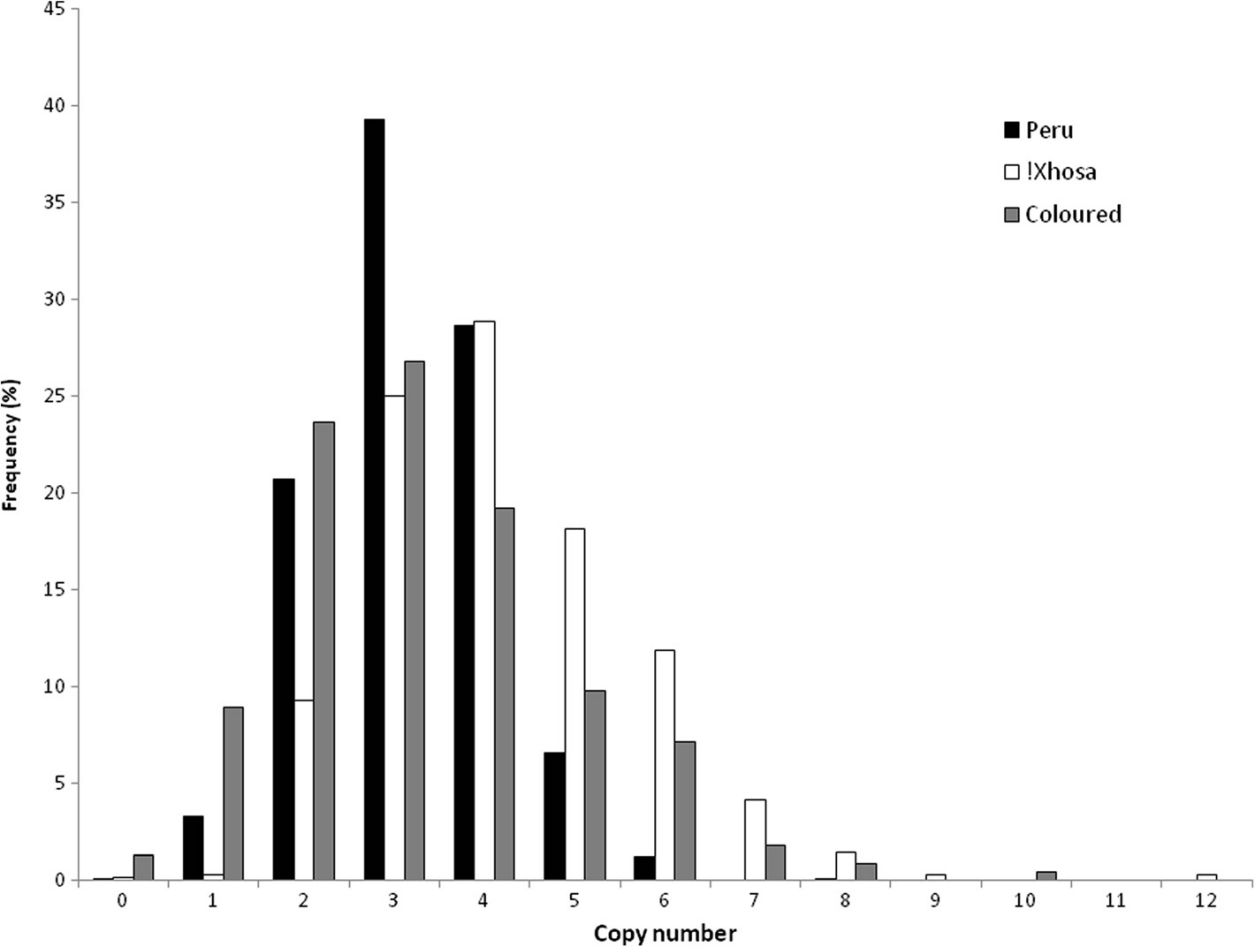
**Copy number distribution within the different populations.** The distribution of copy number values in the different populations with the Peruvian population in black, !Xhosa population in white and Coloured population in grey.

The three populations have different distributions of *CCL3L1*/*CCL4L1* copy numbers, with the !Xhosa population having a significantly different mean to both the Peruvian (p < 0.0001) and Coloured (p < 0.0001) populations (Figure 1).

### Analysis of association between *CCL3L1/CCL4L1* copy number and TB

**Figure 2:**
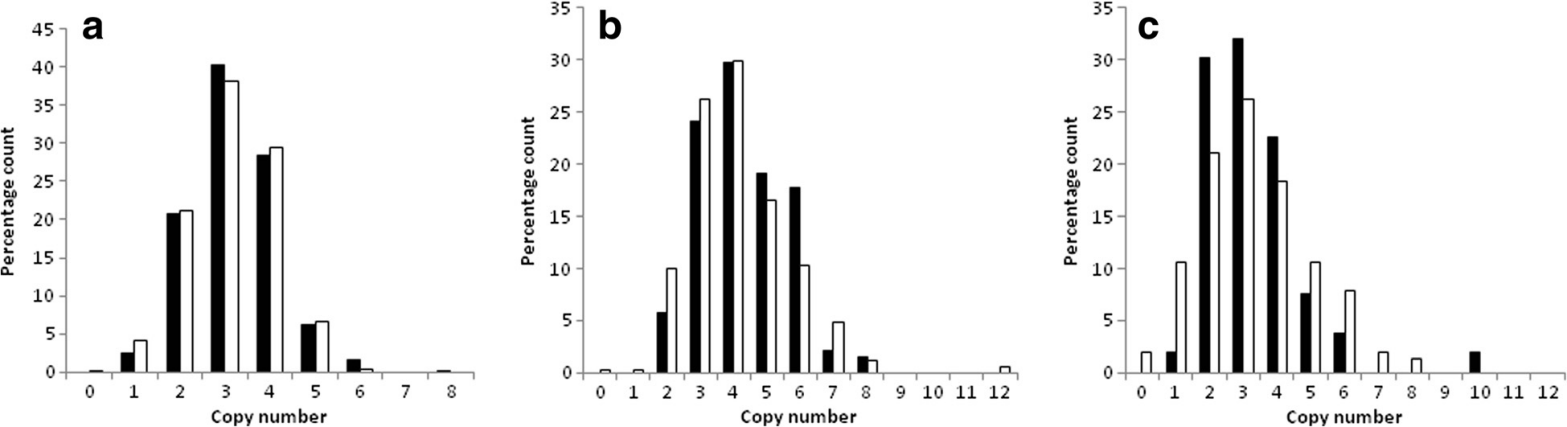
**Copy number distributions between cases and controls within the different populations.** Histogram of the **(a)** Peruvian TB cases (n = 610) and control samples (n = 513), **(b)** !Xhosa paediatric cases (n = 141) and adult control samples (n = 341) and **(c)** Coloured paediatric cases (n = 53) and adult control samples (n = 152). Cases are shown in black and controls in white. There are no significant differences in mean copy number between the cases and controls for any dataset.

For the Peruvian population no significant differences were observed between the control samples (n = 511), with all clinically active TB cases (n = 621) (p = 0.222) (Figure 2a) and then with the subtypes of cases separately; pulmonary (n = 498) (p = 0.279), pleural (n = 79) (p = 0.674), miliary (n = 35) (p = 0.849) or other extrapulmonary (n = 9) (p = n/s) forms of TB. To see whether copy number influences the severity of TB comparison between the pulmonary samples with the pleural, miliary and other extra-pulmonary samples combined was performed, and no significant difference was observed (p = 0.335).

For the !Xhosa population no significant difference in the mean copy number between the paediatric cases (n = 141), and both the paediatric controls (n = 123) (p = 0.841) and the adult controls (n = 341) (p = 0.199) (Figure 2b) was observed. To address whether copy number influences severity differences between pulmonary (n = 55) and extra-pulmonary (n = 82) TB samples were performed but no significant difference was observed (p = 0.939).

For the smaller Coloured population no significant difference in the means between the paediatric cases (n = 53), and both the paediatric controls (n = 13) (p = 0.06) and the adult controls (n = 152) (p = 0.591) (Figure 2c) was observed. To address whether copy number influences severity of TB in the Coloured population differences in the mean copy number between pulmonary (n = 24) and extra-pulmonary (n = 31) TB samples were performed but no significant difference were observed (p = 0.981).

### Analysis of association between *CCR5* genotypes and TB

**Figure 3:**
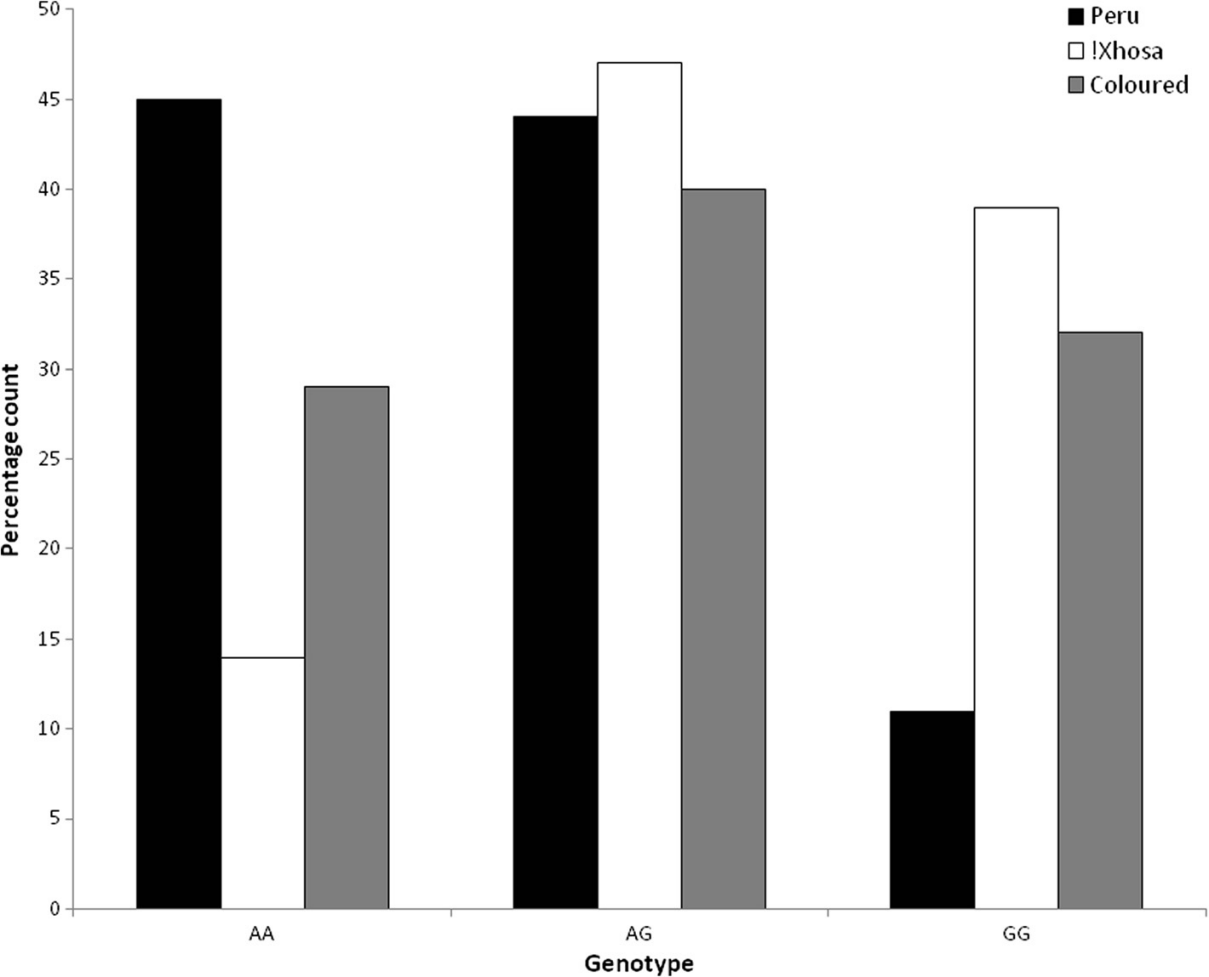
**Distribution of *CCR5 *genotypes in the different populations.** The distribution of *CCR5* genotypes in the different populations, with the Peruvian population in black, !Xhosa population in white and Coloured population in grey.

The allele frequencies of the *CCR5* SNP (rs1799987) differ between the three populations, and significant differences (p < 0.0001) were observed between the frequency in Peru and the frequency of the two South African populations (*A; Peru f = 0.67, !Xhosa f = 0.38, Coloured f = 0.48) (see Figure 3). Case–control analysis with *CCR5* genotype was performed for all three populations between all the cases and controls (both paediatric and adult controls combined for the !Xhosa and Coloured populations) and also between pulmonary TB and other extrapulmonary TB forms. No significant association was observed between *CCR5* genotypes with any of the clinically active TB phenotypes for any population (see Tables 2, 3 and 4).

**Table 2:** **The distributions of *CCR5 *** **genotypes in cases and controls for the Peruvian samples**

**Genotypes**	**Controls**	**All cases**	**Pulmonary**	**Pleural**	**Military**	**Extra pulmonary**
AA	218 (44%)	275 (46%)	218 (45%)	36 (47%)	16 (55%)	5 (56%)
AG	220 (44%)	265 (44%)	218 (45%)	33 (43%)	11 (38%)	3 (33%)
GG	62 (12%)	61 (10%)	50 (10%)	8 (10%)	2 (7%)	1 (11%)

**Table 3:** **The distributions of *CCR5 *** **genotypes in cases and controls for the !Xhosa samples**

**Genotypes**	**Cases**	**Paediatric controls**	**Adult controls**	**All controls**
AA	15 (10%)	22 (18%)	52 (14%)	74 (15%)
AG	79 (53%)	47 (40%)	169 (47%)	216 (45%)
GG	56 (37%)	50 (42%)	144 (39%)	194 (40%)

**Table 4:** **The distributions of *CCR5 *** **genotypes in cases and controls for the coloured samples**

**Genotypes**	**Cases**	**Paediatric controls**	**Adult controls**	**All controls**
AA	17 (29%)	1 (10%)	45 (30%)	46 (28%)
AG	23 (40%)	5 (45%)	59 (40%)	64 (40%)
GG	18 (31%)	5 (45%)	46 (30%)	51 (32%)

### Combined analysis of *CCL3L1* copy number variation and *CCR5* genotype on disease

To investigate the influence of both polymorphisms simultaneously on disease we investigated the distribution of *CCR5* genotypes with *CCL3L1* copy number and performed a logistic regression. There was no clear evidence for an association of combined *CCR5* genotype and copy number with the Peruvian population between all clinically active TB cases and controls (see Additional file 2: Figure S4), and also between control samples and different TB subtypes. For the !Xhosa population there is some suggestion with the logistic regression for an effect of *CCL3L1* copy number when combined with *CCR5* genotype (p = 0.042). The histogram (see Additional file 3: Figure S5) showing the distribution of the raw data however does not obviously support this inference and therefore suggests potentially a type I error (p = 0.126 after Bonferroni correction). There was some suggestion with the Coloured population for an increased proportion of TB cases (14%) presenting with an *AA genotype on a 2-copy background (see Additional file 4: Figure S6), but this is not significant by logistic regression analysis. There was also no evidence for an association of combined *CCR5* genotype and copy number between pulmonary Coloured cases and extra-pulmonary Coloured cases.

## Discussion

Detailed analysis of the raw copy number data generated from the PRT methodology shows the copy number genotyping in the Peruvian population to have a high degree of accuracy in integer copy number prediction, and that the high level of accuracy is consistent with other datasets [28,30]. Whilst the raw copy number data generated for the two South African populations are not as precise as has previously been described for African samples [29], the PRT data and the microsatellite data are consistent in copy number estimation and integer copy number can be assigned with confidence.

This study is the first to investigate susceptibility to clinically active TB in African and Peruvian populations with *CCL3L1*/*CCL4L1* copy number variation. As there is good evidence that strains of *M. tuberculosis* differ geographically, so it is possible that the key factors in the immune response will differ geographically also [36]. There was, thus, the firm potential in this study, with three diverse populations, to identify associations in one population but not any of the other. However, surprisingly the association studies of *CCL3L1*/*CCL4L1* copy number provide no evidence for an association of copy number with clinically active TB in all three populations. A previous study (n = 298) found evidence individuals with *CCL3L1* copy numbers greater than the average (CN = 2) were associated with increased susceptibility to active TB in a Colombian population [24]. However our study could not replicate this observation in Peruvian samples despite the greater sample size. It has previously been reported that higher copy numbers of *CCL3L1*/*CCL4L1* are associated with increased MIP-1α levels and enhanced chemotactic activity [37], which suggests an increased recruitment of immune mediators and potential immunopathology in TB in individuals with higher copy numbers. A recent study by our lab, investigating the consequence of copy number variation at *CCL3L1* did not find significant evidence for an association between increased MIP-1α levels and *CCL3L1* copy number, and actually suggested that variance in expression of the non-copy variable gene *CCL3* is of more biological consequence [38].

There was no evidence provided by this study for an association of TB susceptibility with the functional *CCR5* polymorphism (rs1799987). There is strong linkage disequilibrium across the *CCR5* promoter and recently *CCR5* promoter haplotypes have been studied in relation to TB susceptibility [24]. The polymorphism studied here was included in that analysis along with 6 other polymorphisms, and a *CCR5* promoter haplotype containing the -2459*G allele was shown to be significantly associated with TB, independent of age and sex and *CCL3L1* copy number.

Interestingly there was a marked difference in *CCR5* allele frequencies between the different populations, such that in Peru the high expression allele predominates, whereas in the !Xhosa population it is the low expression allele that is in the majority. This has implications in the immune response to other infectious disease, including HIV infection which uses CCR5 as a co-receptor for cell entry [39,40]. It is also possible that as the CCR5 low expression allele predominates in Africa this may play a role, albeit minor, in the maintenance of higher *CCL3L1*/*CCL4L1* copy numbers in African samples to compensate.

The lack of association observed with the *CCR5* polymorphism may be due to the fact that CCR5 function can be compensated for by other receptors with overlapping specificities. MIP-1α has been shown to also functionally interact with receptors CCR1 and CCR3, both of which are present on numerous immune cells. Mice that lack *CCR5* can still induce a Th1 response, control infection and recruit immune cells to form a granuloma when infected with *M. tuberculosis*[41], and can control infection with L. donovani, another macrophage pathogen [42]. Furthermore, individuals who are homozygous for *CCR5Δ32*, thus having no functioning CCR5 receptor, appear to be immunologically normal [21].

The three populations analysed here provided no evidence for any associations with clinically active TB susceptibility when investigating variations in *CCR5* and *CCL3L1* copy number together. The correlation of variants in the chemokine-chemokine receptor pair with TB susceptibility may have been anticipated from functional analyses, but our data does not support this. A recent observation identified that haplotypes in *CCR5* associated with increased expression of CCR5 are associated with TB susceptibility in individuals with 2 copies or more of *CCL3L1*[24]. Our understanding of the precise role of cytokines in TB infection remains limited (reviewed in [43]) and there is much compensation and overlap of roles due to the complexity of the system. To conclude, whilst functional studies have provided evidence that both MIP-1α and CCR5 may play a role in TB susceptibility, this study of genetic variation in *CCL3L1* and *CCR5* does not provide additional evidence to support these roles.

## Competing interests

The author’s have no personal or financial interests to declare.

## Authors’ contributions

DC participated in the study design, coordinated the study, performed all copy number typing, all CCR5 genotyping, statistical analysis, and drafted the manuscript. CT collected all the Peruvian samples and phenotype data and generated the DNA from the Peruvian samples. JG collected the South African DNA samples and phenotypes. ML collected the South African DNA samples and phenotypes and helped to draft the manuscript. BE collected the South African DNA samples and phenotypes and helped to draft the manuscript. SA collected the South African DNA samples and phenotypes and helped to draft the manuscript. MAS participated in the study design and helped to draft the manuscript. JALA participated in the study design and helped to draft the manuscript. All authors approved the final version of the manuscript.

## Pre-publication history

The pre-publication history for this paper can be accessed here:

http://www.biomedcentral.com/1471-2350/15/5/prepub

## Supplementary Material

Additional file 1 **A detailed description of how integer copy number was assigned.** Figure S1 Distribution of average calibrated copy number values for the 1132 genotyped Peruvian samples. Figure S2 Distribution of (a) average calibrated copy number values and (b) with additional microsatellite data included (represented by a different colour for each integer) for the 493 genotyped South African adult samples, comprising 341 !Xhosa samples and 152 Coloured samples. Figure S3 Distribution of (a) average calibrated copy number values and (b) with additional microsatellite data included (represented by a different colour for each integer) for the 343 genotyped South African paediatric samples comprising 264 !Xhosa samples, 69 Coloured samples and 10 samples of unknown ethnicity. Click here for file

Additional file 2: Figure S4 Histogram of all the Peruvian case and control data stratified by both copy number and CCR5 genotype. The histogram shows no significant difference in the distribution of CCR5 genotype with CCL3L1/CCL4L1 copy number between the cases and controls. Click here for file

Additional file 3: Figure S5 Histogram of all the !Xhosa case and control data stratified by both copy number and CCR5 genotype. The histogram shows no significant difference in the distribution of CCR5 genotype with CCL3L1/CCL4L1 copy number between the cases and controls. Click here for file

Additional file 4: Figure S6 Histogram of all the Coloured case and control data stratified by both copy number and CCR5 genotype. The histogram shows some suggestion of a distortion in the distribution of AA genotype on a 2-copy background in the cases. Click here for file
