# Supplementary material for: CCL3L1 copy number, CCR5 genotype and susceptibility to tuberculosis
Source: BMC Med Genet. 2014 Jan 9;15:5. doi: 10.1186/1471-2350-15-5 (PMC3897992; doi:10.1186/1471-2350-15-5)
Supplement: Additional file 1 — A detailed description of how integer copy number was assigned. Figure S1 Distribution of average calibrated copy number values for the 1132 genotyped Peruvian samples. Figure S2 Distribution of (a) average calibrated copy number values and (b) with additional microsatellite data included (represented by a different colour for each integer) for the 493 genotyped South African adult samples, comprising 341 !Xhosa samples and 152 Coloured samples. Figure S3 Distribution of (a) average calibrated copy number values and (b) with additional microsatellite data included (represented by a different colour for each integer) for the 343 genotyped South African paediatric samples comprising 264 !Xhosa samples, 69 Coloured samples and 10 samples of unknown ethnicity. [file 1471-2350-15-5-S1.doc]

**Supplementary results**

The paralogue ratio test (PRT) was used to genotype the copy number of the *CCL3L1*/*CCL4L1* copy variable region in a total of 1150 samples of Peruvian origin and 899 samples of South African origin, of which were 532 were adults and 367 were children.

For the Peruvian samples the PRT assays assigned concordant measurements of copy number to within 0.5 of the inferred integer value for 73% of samples (839/1150), and to within 0.75 of the inferred integer for 96% of samples (1108/1150). Microsatellite typing was carried out for 25 samples that showed some discordance in the PRT calling, this allowed confident copy number calling for a further 24 samples, leaving 1 sample for which the copy number cannot be confidently resolved and these have therefore been excluded from the association analysis. There are 17 samples for which the PRT failed, likely due to low DNA concentrations, leaving a total of 1132 Peruvian samples. The distribution of the unrounded calibrated copy numbers for the Peruvian population is shown in supplementary figure 1, clearly demonstrates PRT values clustering around the inferred integers.

The overall standard deviation (normalised for copy number) of the full Peruvian dataset was 0.069, which is consistent with previously typed datasets [1-3]. The mean and standard deviation, normalised standard deviations and predicted probability of error for the full dataset are shown in supplementary table 1. The data show that the means lie within 0.1 of the corresponding integer and that the standard deviations are sufficiently low that the probability of integer error is also small.

For each South African sample three independent triplex PCR reactions and two microsatellites assays were combined into a single capillary for each sample. Mean unrounded calibrated copy numbers were generated for each PRT system (termed CCL3C, CCL4A and LTR61A), as well as a single mean value, and then compared to the integer copy number predicted from the microsatellite data [2]. As the adult and paediatric samples were collected separately then copy number measurement and analysis is conducted separately to avoid any differential bias that may be introduced due to the differences in DNA quality.

For the South African adult samples the three independent PRT assays assigned concordant measurements of copy number to within 0.5 of the inferred integer value for 20% of samples (102/532), and to within 0.75 of the inferred integer for 64% of samples (343/532). The distribution of the raw data is shown in supplementary figure 2a, inclusion of the predicted integer copy number value generated from the microsatellite data adds support for the predicted integer copy number values from the PRT (see supplementary figure 2b). In the majority of cases (95%) the integer copy number value predicted from the microsatellite data agreed with that from the PRT data (491/532). There were 32 cases where the PRT measurements and the microsatellites were in disagreement such that an integer copy number could not be concluded, these samples are not in any further analysis. There were 7 failed samples, giving a total of 493 typed adult samples, comprising of 341 !Xhosa samples and 152 Coloured samples.

The overall standard deviation (normalised for copy number) of the full adult dataset was 0.084, and the mean and standard deviation, normalised standard deviations and predicted probability of error for the full dataset are shown in supplementary table 2.

For the South African paediatric samples the three independent PRT assays assigned concordant measurements of copy number to within 0.5 of the inferred integer value for 27% of samples (101/367), and to within 0.75 of the inferred integer for 46% of samples (169/367). The distribution of the raw data is shown in supplementary figure 3a, inclusion of the predicted integer copy number value generated from the microsatellite data adds support for the predicted integer copy number values from the PRT (see supplementary figure 3b). In the majority of cases (93%) the integer copy number value predicted from the microsatellite data agreed with that from the PRT data (343/367). There were 24 cases where the PRT measurements and the microsatellites were in disagreement such that an integer copy number could not be concluded, these samples are not in any further analysis. Thus there is a final total of 343 paediatric samples genotyped, consisting of 264 !Xhosa samples and 69 Coloured samples and 10 samples of unknown ethnicity or phenotype and which are not in any further analysis.

The overall standard deviation (normalised for copy number) of the paediatric dataset was 0.093, and the mean and standard deviation, normalised standard deviations and predicted probability of error for the full dataset are shown in supplementary table 3.

| Supplementary Table 1; Mean, deviations and error rates for the calibrated copy number values in the Peruvian population | | | | | | |
| --- | --- | --- | --- | --- | --- | --- |
| Copy Number | No | Mean | Standard deviation | Normalised Standard deviation | Specific deviation | Predicted error rate |
| 0 | 1 | 0.242 |  |  |  |  |
| 1 | 36 | 1.134 | 0.129 | 0.129 | 0.188 | 2.4x10^-3^ |
| 2 | 237 | 2.080 | 0.208 | 0.074 | 0.119 | 2.5x10^-2^ |
| 3 | 445 | 3.024 | 0.171 | 0.057 | 0.099 | 3.9x10^-3^ |
| 4 | 327 | 3.971 | 0.187 | 0.047 | 0.095 | 8.3x10^-3^ |
| 5 | 73 | 4.952 | 0.271 | 0.054 | 0.123 | 6.9x10^-2^ |
| 6 | 12 | 5.869 | 0.231 | 0.038 | 0.105 | 5.8x10^-2^ |
| 7 |  |  |  |  |  |  |
| 8 | 1 |  |  |  |  |  |

| Supplementary Table 2; Mean, deviations and error rates for average calibrated copy number values from the PRT data for the South African adult samples | | | | | | |
| --- | --- | --- | --- | --- | --- | --- |
| Copy Number | No | Mean | Standard deviation | Normalised Standard deviation | Specific deviation | Predicted error rate |
| 0 | 4 |  |  |  |  |  |
| 1 | 17 | 0.799 | 0.172 | 0.172 | 0.269 | 0.041 |
| 2 | 64 | 1.741 | 0.168 | 0.084 | 0.219 | 0.076 |
| 3 | 131 | 2.669 | 0.232 | 0.077 | 0.234 | 0.233 |
| 4 | 128 | 3.654 | 0.309 | 0.077 | 0.232 | 0.312 |
| 5 | 76 | 4.622 | 0.349 | 0.069 | 0.231 | 0.369 |
| 6 | 44 | 5.553 | 0.403 | 0.067 | 0.247 | 0.457 |
| 7 | 21 | 6.491 | 0.359 | 0.051 | 0.282 | 0.512 |
| 8 | 7 | 7.349 | 0.305 | 0.038 | 0.270 | 0.689 |
| 9 |  |  |  |  |  |  |
| 10 |  |  |  |  |  |  |
| 11 |  |  |  |  |  |  |
| 12 | 1 | 12.2 |  |  |  |  |

| Supplementary Table 3; Mean, deviations and error rates for average calibrated copy number values from the PRT data for the South African paediatric samples | | | | | | |
| --- | --- | --- | --- | --- | --- | --- |
| Copy Number | No | Mean | Standard deviation | Normalised Standard deviation | Specific deviation | Predicted error rate |
| 0 |  |  |  |  |  |  |
| 1 | 5 | 0.897 | 0.197 | 0.197 | 0.228 | 0.023 |
| 2 | 44 | 1.948 | 0.190 | 0.095 | 0.139 | 0.011 |
| 3 | 85 | 2.989 | 0.298 | 0.099 | 0.172 | 0.093 |
| 4 | 94 | 4.064 | 0.347 | 0.087 | 0.177 | 0.157 |
| 5 | 61 | 5.074 | 0.412 | 0.082 | 0.187 | 0.232 |
| 6 | 42 | 6.151 | 0.527 | 0.088 | 0.224 | 0.362 |
| 7 | 11 | 7.083 | 0.479 | 0.069 | 0.183 | 0.304 |
| 8 | 5 | 7.956 | 0.901 | 0.113 | 0.319 | 0.579 |
| 9 | 2 | 9.654 | 0.303 | 0.034 | 0.325 | 0.695 |
| 10 | 1 | 9.753 |  |  |  |  |

**
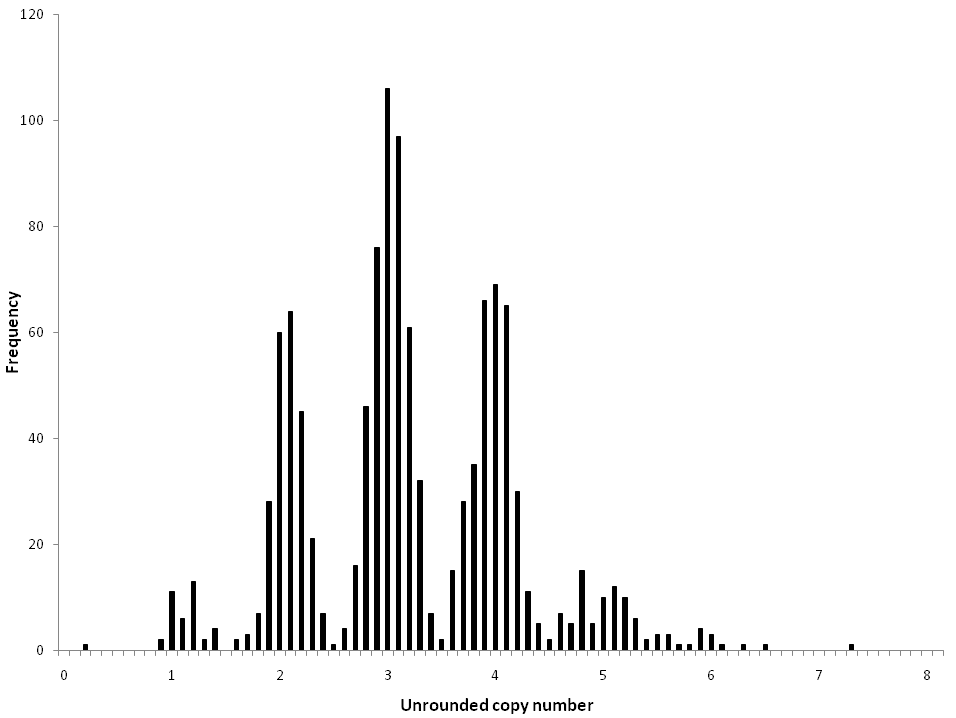
**

**
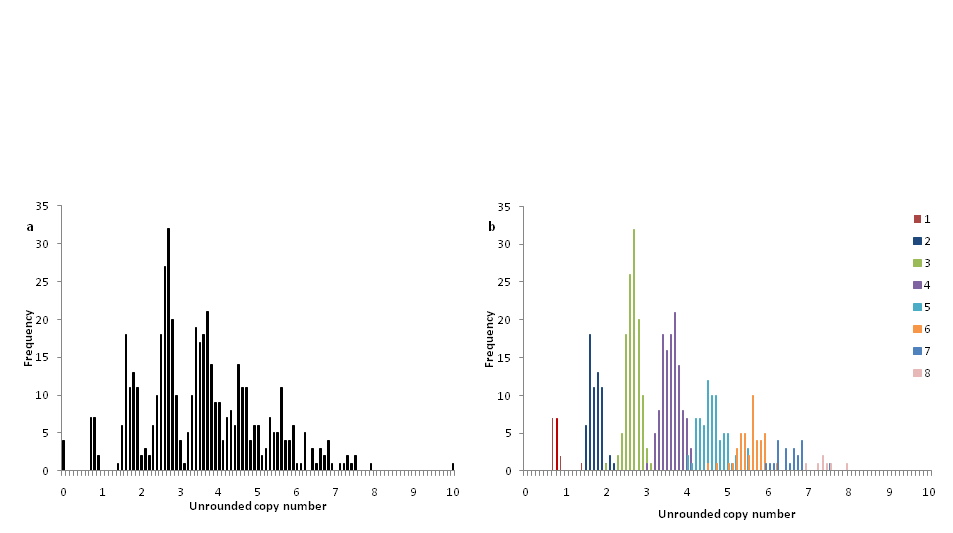
**

**
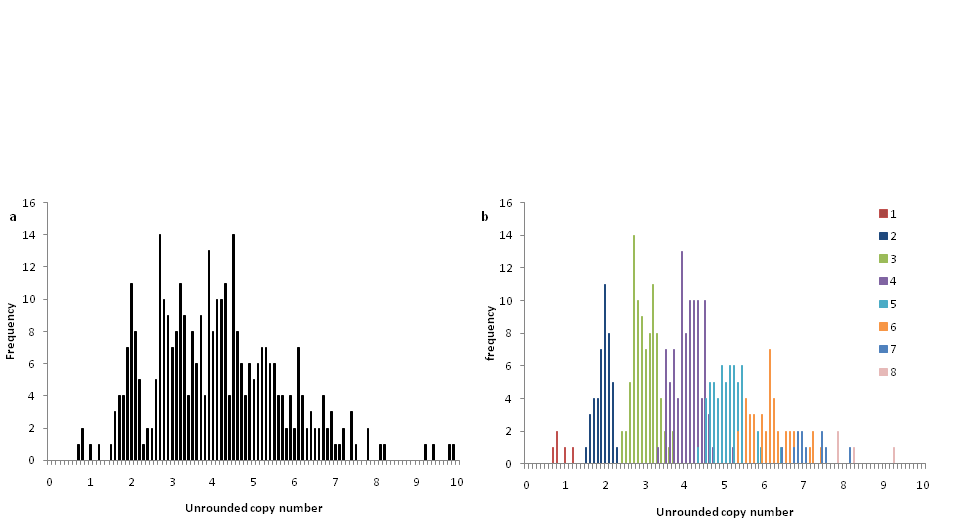
**

**
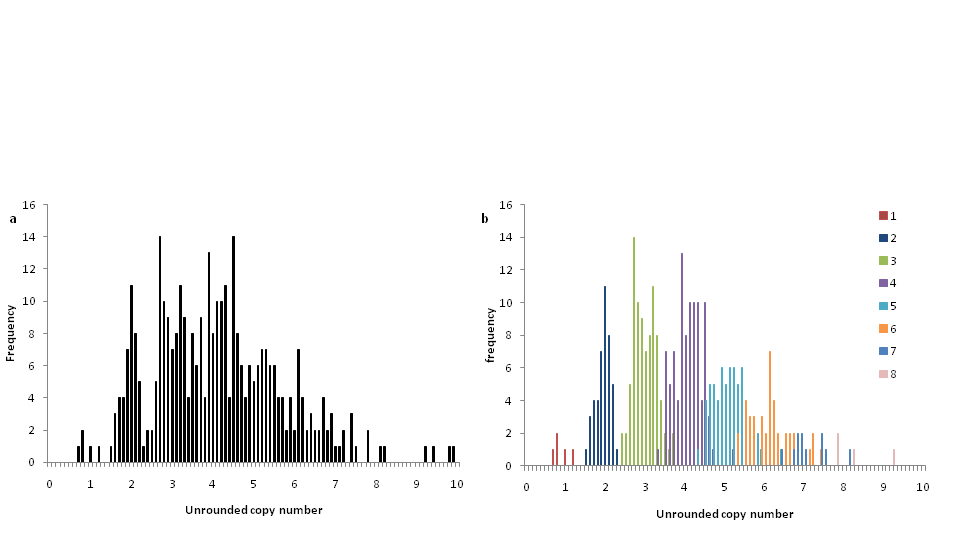
**

**References**

1. Walker S, Janyakhantikul S, Armour JAL: **Multiplex Paralogue Ratio Tests for accurate measurement of multiallelic CNVs**. *Genomics* 2009, **93**(1):98-103.

2. Carpenter D, Rooth I, Armour JAL, Shaw MA: **CCL3L1 copy number and susceptibility to malaria**. *Infection, Genetics and Evolution* 2012, **12**:1147-1154.

3. Carpenter D, McIntosh R, Pleass R, Armour JAL: **Functional effects of CCL3L1 copy number**. *Genes and Immunity* 2012, **13**:374-379.
