# Supplementary figures and images for: CCL3L1 copy number, CCR5 genotype and susceptibility to tuberculosis
Source: BMC Med Genet. 2014 Jan 9;15:5. doi: 10.1186/1471-2350-15-5 (PMC3897992; doi:10.1186/1471-2350-15-5)

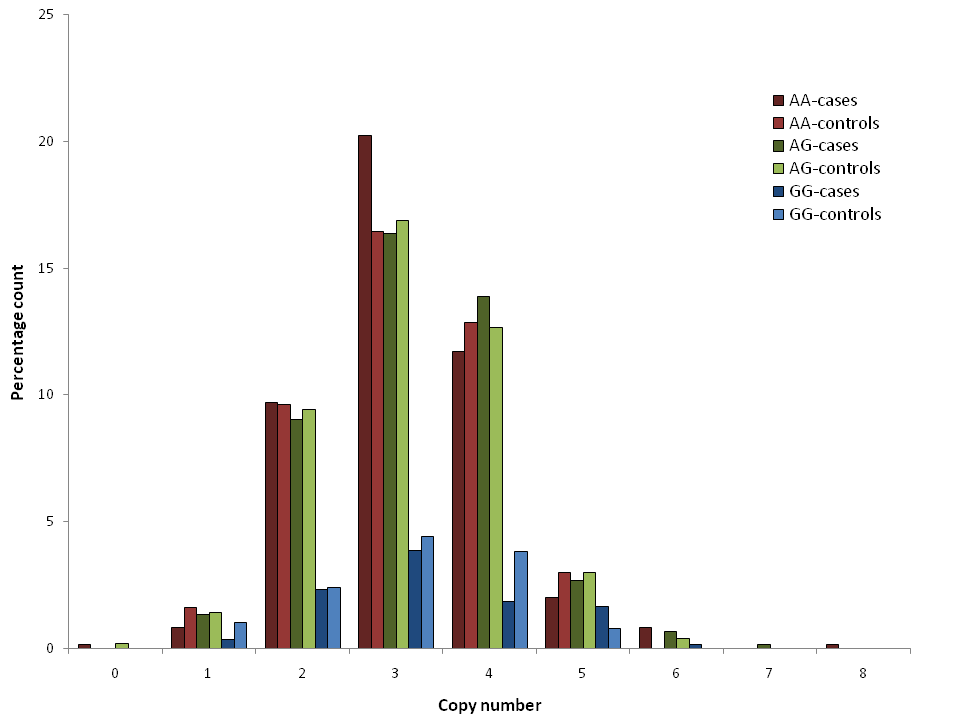

Supplement: Additional file 2: Figure S4 — Histogram of all the Peruvian case and control data stratified by both copy number and CCR5 genotype. The histogram shows no significant difference in the distribution of CCR5 genotype with CCL3L1/CCL4L1 copy number between the cases and controls. [file 1471-2350-15-5-S2.tiff]

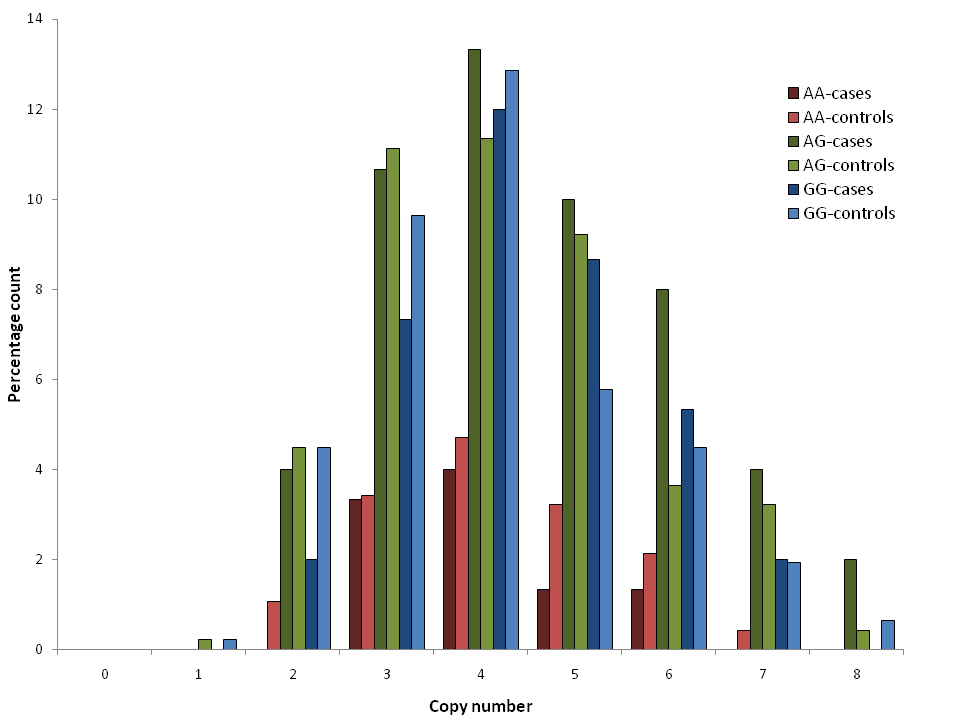

Supplement: Additional file 3: Figure S5 — Histogram of all the !Xhosa case and control data stratified by both copy number and CCR5 genotype. The histogram shows no significant difference in the distribution of CCR5 genotype with CCL3L1/CCL4L1 copy number between the cases and controls. [file 1471-2350-15-5-S3.tiff]

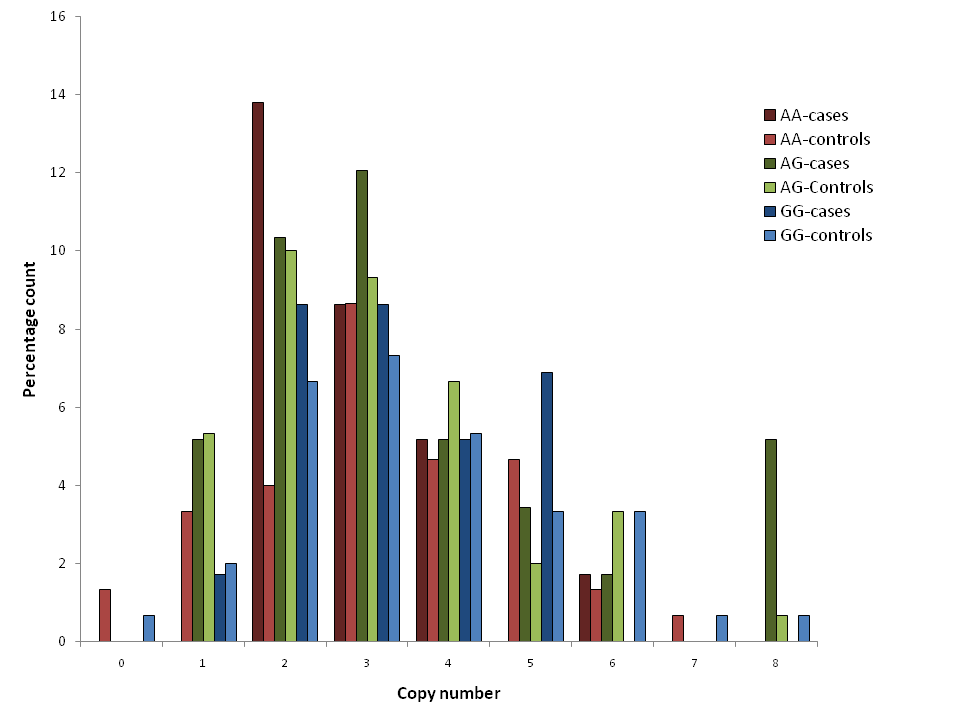

Supplement: Additional file 4: Figure S6 — Histogram of all the Coloured case and control data stratified by both copy number and CCR5 genotype. The histogram shows some suggestion of a distortion in the distribution of AA genotype on a 2-copy background in the cases. [file 1471-2350-15-5-S4.tiff]
